# The morphology of the femoral posterior condyle affects the external rotation of the femur

**DOI:** 10.1186/s40634-023-00686-w

**Published:** 2023-11-25

**Authors:** Hiroki Hijikata, Osamu Tanifuji, Tomoharu Mochizuki, Takashi Sato, Satoshi Watanabe, Ryota Katsumi, Sho Hokari, Hiroyuki Kawashima

**Affiliations:** 1 Division of Orthopaedic Surgery, Department of Regenerative and Transplant Medicine, Niigata University Graduate School of Medicine and Dental Science, 1-757 Asahimachi-Dori Chuo-Ku, Niigata, Japan; 2 Department of Orthopaedic Surgery, Niigata Medical Center, Niigata, Japan; 3 Department of Orthopaedic Surgery, Niigata Prefectural Shibata Hospital, Niigata, Japan

**Keywords:** Knee motion, Motion analysis, Femoral rotation, Femoral morphology, tibial morphology, Alignment of lower extremities, Three-dimensional to two-dimensional registration technique

## Abstract

**Purpose:**

The purpose of this study was to identify factors related to the external rotation of the femur during knee flexion.

**Methods:**

Three-dimensional (3D) digital models of the femur and tibia were reconstructed from computed tomography images of 41 healthy Japanese subjects. Thirteen parameters related to femoral and tibial morphology and alignment of the lower extremities were evaluated, including the inclination angle of the posterior lateral and medial femoral condyles, the ratio of the medial and lateral posterior condyle radii approximated as spheres, the spherical condylar angle, the posterior condylar angle, the medial and lateral posterior tibial slope, the difference of medial and lateral posterior tibial slope, the tibiofemoral rotation angle, the 3D femorotibial angle, the 3D hip-knee-ankle angle, and the passing point of the weight-bearing line (medial–lateral and anterior–posterior). The rotation angle of the femur relative to the tibia during squatting was investigated using a 3D to 2D image matching technique and the relationships with the13 parameters were determined.

**Results:**

The femur externally rotated substantially up to 20° of knee flexion (9.2° ± 3.7°) and gently rotated after 20° of knee flexion (12.8° ± 6.2°). The external rotation angle at 20°–120° of knee flexion correlated with the spherical condylar angle, the tibiofemoral rotation angle and the inclination angle of the posterior medial condyles (correlation coefficient; 0.506, 0.364, 0.337, respectively).

**Conclusion:**

The parameter that was most related to the external rotation of the femur during knee flexion was the spherical condylar angle.

**Level of Evidence:**

IV

## Introduction

Knowledge of the relative motion between the femur and tibia of normal knees is important for understanding the pathology of various knee-related diseases. Motion analyses of normal knees have been described in many reports. In a normal knee, it is known that the medial side shows stable, whereas the lateral side exhibits significant posterior translation throughout knee flexion. As a result, the femur exhibits external rotation relative to the tibia, referred to as “medial pivot motion” [1–6]. Knees with medial osteoarthritis display less femoral external rotation compared to normal knees [7, 8]. The magnitudes of axial rotation did not restore after total knee arthroplasty (TKA) when compared to normal knees during a deep knee bend [9]. Furthermore, even in the normal knee, there are individual differences in the amount of femoral rotation. However, the factors that cause medial pivot motion are not clear. In recent years, the normal morphological features of the femoral posterior condyles were characterized, and it was shown that the medial condyle was almost vertical, whereas the lateral condyle was tilted medially. [10]. This asymmetrical inclination between the medial and lateral condyles or any other shape of the femoral condyle may affect the external rotation of the femur relative to the tibia throughout knee flexion. Additionally, shape of tibial condyle and lower extremity alignment may influence external rotation of the femur. In particular, the tilt of the lateral posterior condyle was thought to induce medial pivot motion by inducing the lateral condyle to move in a medial direction. The purpose of this study was to identify factors related to the external rotation of the femur during knee flexion using three-dimensional (3D) analysis. It was hypothesized that the degree of external rotation of the femur relative to the tibia correlates with the inclination angle of the lateral posterior condyle of the femur. Identification of factors related to femoral external rotation relative to the tibia is considered to be reference data for elucidating the pathological condition of a disease such as osteoarthritis of the knee. Furthermore, these findings could influence TKA implant design.

### Materials and methods

This study was performed according to the protocol approved by the Investigational Review Boards of our institutions (Niigata University, 2017–0006). All subjects provided informed consent before participating in this study. The inclusion criteria of this study were subjects who had no knee complaints or history of trauma to the knee. The exclusion criteria of this study were subjects who had abnormalities in physical tests (positive McMurray’s test or joint line tenderness) or abnormalities in radiographic examination (osteophyte around the knee or degenerative change at the hip joint) or could not flex their knees until 120 degrees in squatting during the motion analysis.

First, a total of 62 elderly Japanese volunteers with no knee complaints were recruited prospectively. The doctors evaluated their general and lower extremity conditions using physical tests and radiographies, and 7 subjects were excluded because they had abnormal conditions with radiographic knee osteoarthritis (OA). Fourteen of the remaining 55subjects were excluded because they could not flex their knees until 120 degrees in squatting during the motion analysis. Finally, 41 knees were enrolled in this study with motion analysis of knee flexion.

### Constructing a coordinate system of the femur and tibia

**Fig. 1:**
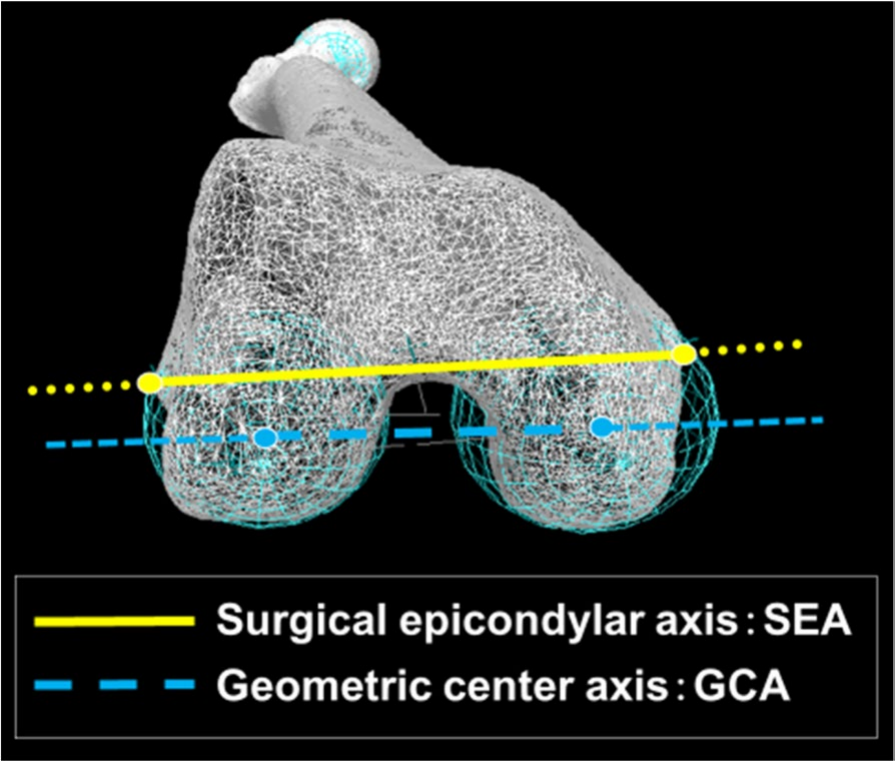
Surgical epicondylar axis (SEA) and the geometric center axis (GCA). The SEA was constructed using the peak point of the lateral epicondyle and the fossa between the anterior peak and the posterior peak on the medial epicondyle. The GCA is the line connecting the centers of the spheres representing the medial and lateral femoral posterior condyles

Computed tomography (CT) (Canon Medical Systems, Tochigi, Japan) scans of bilateral femurs and tibias were obtained for all subjects, with a 1 mm slice thickness. A 3D digital model of the femur and tibia was reconstructed from CT data using 3D visualization and modeling software (Zed-View®; LEXI, Tokyo, Japan). The anatomic coordinate systems were established by referencing several bony landmarks according to the definitions reported by Sato et al. [11]. The femoral x-axis was defined as the line connecting the centers of the spheres representing the medial and lateral posterior femoral condyles (positive laterally). These spheres were constructed by digitizing four points along the contours of the medial and lateral femoral posterior condyles. The origin of the femoral anatomic coordinate system was defined as the midpoint between the centers of these posterior condylar spheres. The femoral z-axis was perpendicular to the x-axis in a plane formed by the x-axis and a line connecting the femoral origin and the center of the femoral head (positive superiorly). The femoral y-axis was defined as the cross product of the z-axis and x-axis (positive anteriorly). The tibial z-axis was defined as a line connecting the midpoint of the tibial eminence and the midpoint of the medial and lateral top of the talar dome. The tibial y-axis (positive anteriorly) was defined as a line drawn perpendicularly from the mediolateral center of the posterior cruciate ligament insertion to the z-axis. The tibial x-axis was defined as the cross product of the z-axis and y-axis. The xy plane in this coordinate system was defined as the tibial axial plane. Using previously applied methods [12–14], the medial and lateral posterior femoral condyles were approximated as spheres that best matched the condyle geometries to define the geometric center axis (GCA). The transepicondylar axis was defined as the surgical epicondylar axis (SEA) (Fig. 1).

### Assessment of bone morphology

**Fig. 2:**
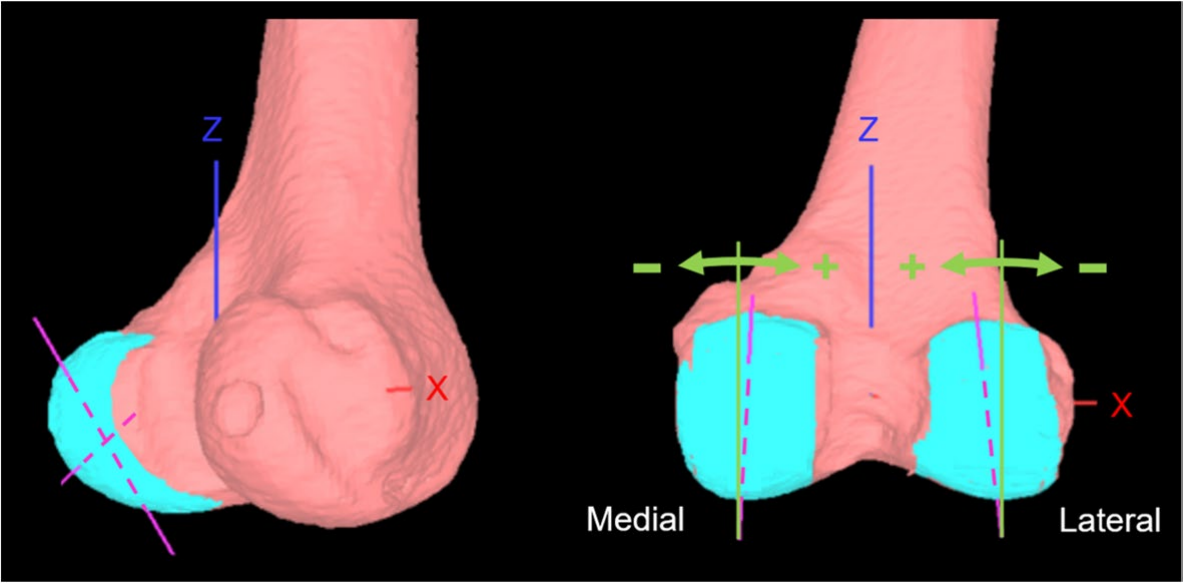
Inclination angle of the posterior condyles. The articular surface of posterior condyles was selected and extracted. The axis of the ellipsoid approximating the posterior condyle was calculated and projected onto the coronal (xz) plane of the femoral coordinate system

The inclination angle of the posterior condyles was determined as described below, according to the definitions reported by Hokari et al. [10]. The 3D models of the femur, including the coordinate systems, were entered into the original software and only the articular surface of the posterior condyles was selected and extracted. In the coordinate system, the area of the articular surface of the posterior condyles was defined as the upper border of the posterior condyles to the most distal level of the femur. The extracted articular surface was approximated as an ellipsoid. The long axis of this approximated ellipsoid was projected onto the coronal plane of the femoral coordinate system and defined as the inclination of the articular surface. An inclination tilting towards the proximal intercondylar fossa was defined as positive in both the medial and lateral condyles (Fig. 2). To　assess inter- and intra-observer reliability, all subjects were measured twice with a 6-week interval by two observers. The intra- and inter-observer reliabilities were assessed by intra-class correlation coefficients (ICC). ICC estimates and their 95% confident intervals were calculated using statistical software (IBM SPSS statistical package version 21; IBM Corp., Armonk, NY, USA) based on a mean-rating (k = 2), absolute-agreement, 2-way mixed-effects model. ICC (1, 2) of the first observer were 0.982 (95%CI:0.974—0.987) and 0.987 (95%CI:0.982—0.991), ICC (1, 2) of the second observer were 0.981 (95%CI:0.976—0.988) and 0.991 (95%CI:0.987—0.994), and the ICC (3, 1) between two observers were 0.975 (95%CI:0.965—0.983) and 0.967 (95%CI:0.953—0.977), respectively.

**Fig. 3:**
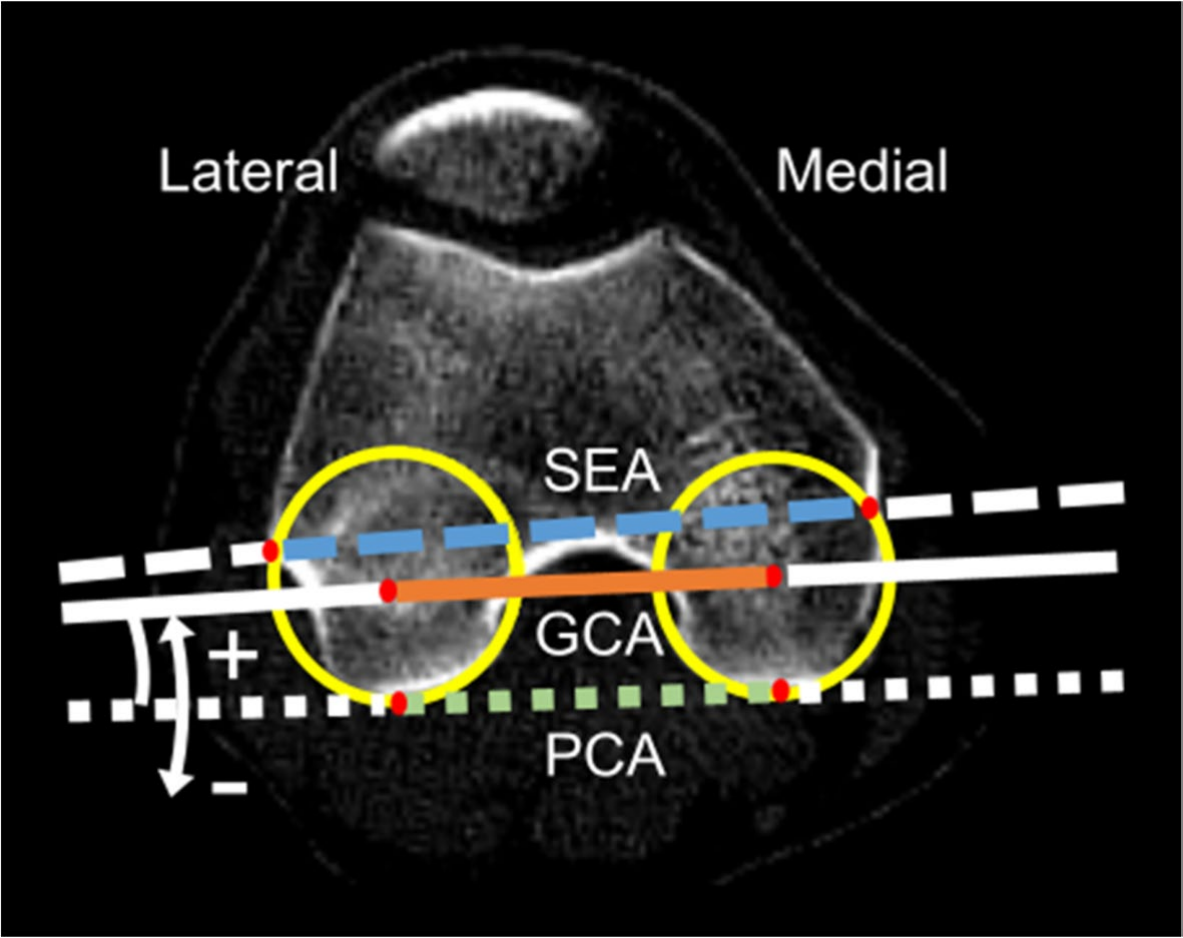
Geometric center axis (GCA), surgical epicondylar axis (SEA), and posterior condylar axis (PCA) in the axial (xy) plane of the femur. The spherical and posterior condylar angles were measured in the axial (xy) plane. The spherical condylar angle was deemed positive when the GCA was rotated externally more than the PCA. The posterior condylar angle was deemed positive when the SEA was rotated externally more than the PCA

The two femoral condyle angles were measured. The spherical condylar angle was defined as the angle between the posterior condylar axis (PCA) and the GCA. The PCA was defined as the tangential line on both the medial and lateral posterior condyles. The other angle was posterior condylar angle defined as the angle between the PCA and the SEA. The GCA, SEA and PCA were projected onto the axial (xy) plane of the femur (Fig. 3).

**Fig. 4:**
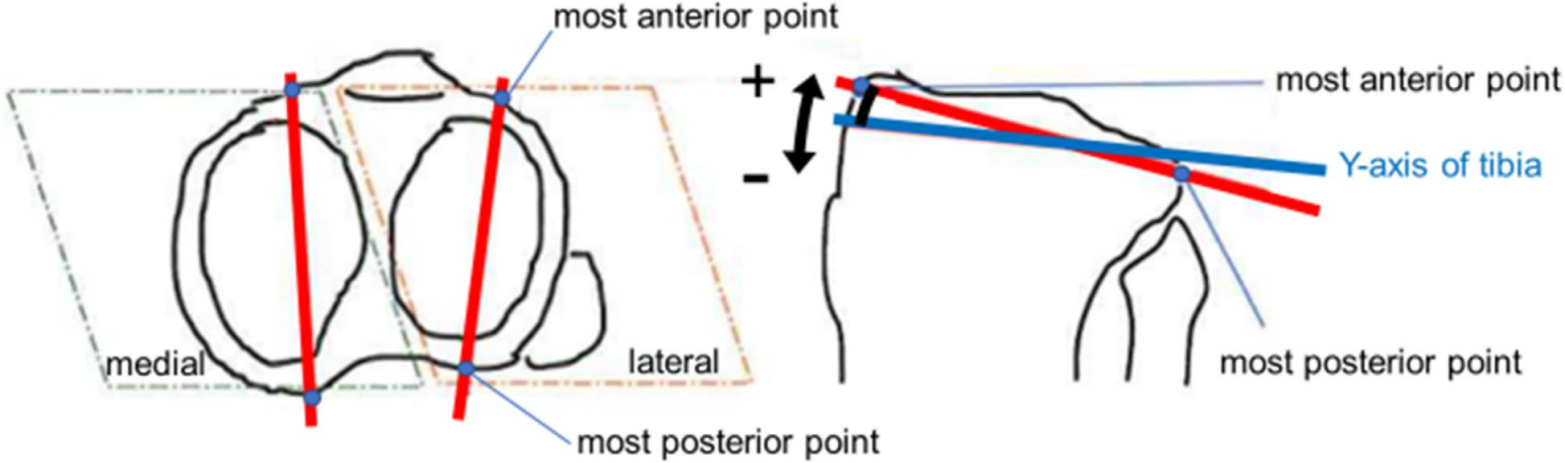
Medial and lateral Posterior Tibial Slope (PTS). Medial and lateral PTS was deemed positive when the line connecting the most anterior point to the most posterior point on the medial and lateral tibial joint surface tilted more posterior than tibial y-axis

The tibial angles were measured on both sides. The medial posterior tibial slope (PTS) was defined as the angle between the line connecting the most anterior to the most posterior point on the medial tibial joint surface and tibial y-axis. The lateral PTS was defined as the angle between the line connecting the most anterior to the most posterior point on the lateral tibial joint surface and tibial y-axis. The line connecting the most anterior to the most posterior point on the medial and lateral tibial joint surface was projected onto the sagittal (yz) plane of the tibia (Fig. 4). The difference in PTS between the medial and lateral sides was also measured. The difference between medial and lateral PTS was defined as positive when the medial PTS tilted more posteriorly than lateral PTS.

### Alignment of the lower extremities

**Fig. 5:**
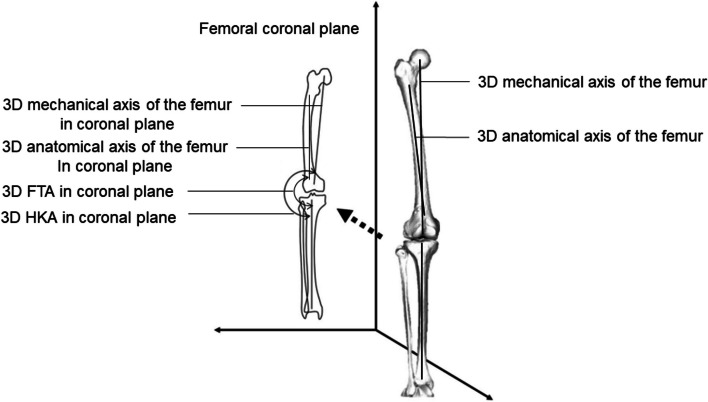
3-dimensional (3D) femorotibial angle (3DFTA) and the 3D hip-knee-ankle angle (3DHKA). The 3D anatomical and mechanical axes of the femur and tibia were defined in a 3D space and projected onto the coronal (xz) plane of the femur. The 3D mechanical axis of the femur was defined as the line connecting the center of the femoral head and the midpoint of the spheres representing the medial and lateral posterior femoral condyles. The 3D mechanical axis of the tibia was defined as the line connecting the midpoint between the medial and lateral eminences of the tibial spines with the center of the ankle joint

Several lower extremity alignment parameters were measured in the weight-bearing (WB) position. WB biplane computed radiography images of the entire lower extremities were obtained, with the knee fully extended and toes in the neutral position. The 3D assessment of the lower extremity alignment was conducted using 3D to 2D image registration techniques by the 3D lower extremity alignment assessment system (Knee CAS; LEXI, Inc., Tokyo, Japan) [15–18]. The tibiofemoral rotation angle was defined as the angle between the femoral x-axis and the tibial x-axis in the xy plane of the femur. The tibiofemoral rotation angle was considered positive when the tibial x-axis was rotated more externally than the femoral x-axis. The 3D femorotibial angle (3DFTA) in the coronal plane was defined as the angle between the anatomical axes of the femur and tibia in the xz femur plane. The 3D hip-knee-ankle angle (3DHKA) in the coronal plane was defined as the angle between the 3D mechanical axes of the femur and tibia in the xz femur plane. The 3DHKA in the coronal plane is a more accurate varus–valgus alignment parameter than the common two-dimensional HKA [15]. The 3DFTA and 3DHKA in the coronal plane indicate that the larger angles are associated with a more varus tibiofemoral alignment (Fig. 5).

**Fig. 6:**
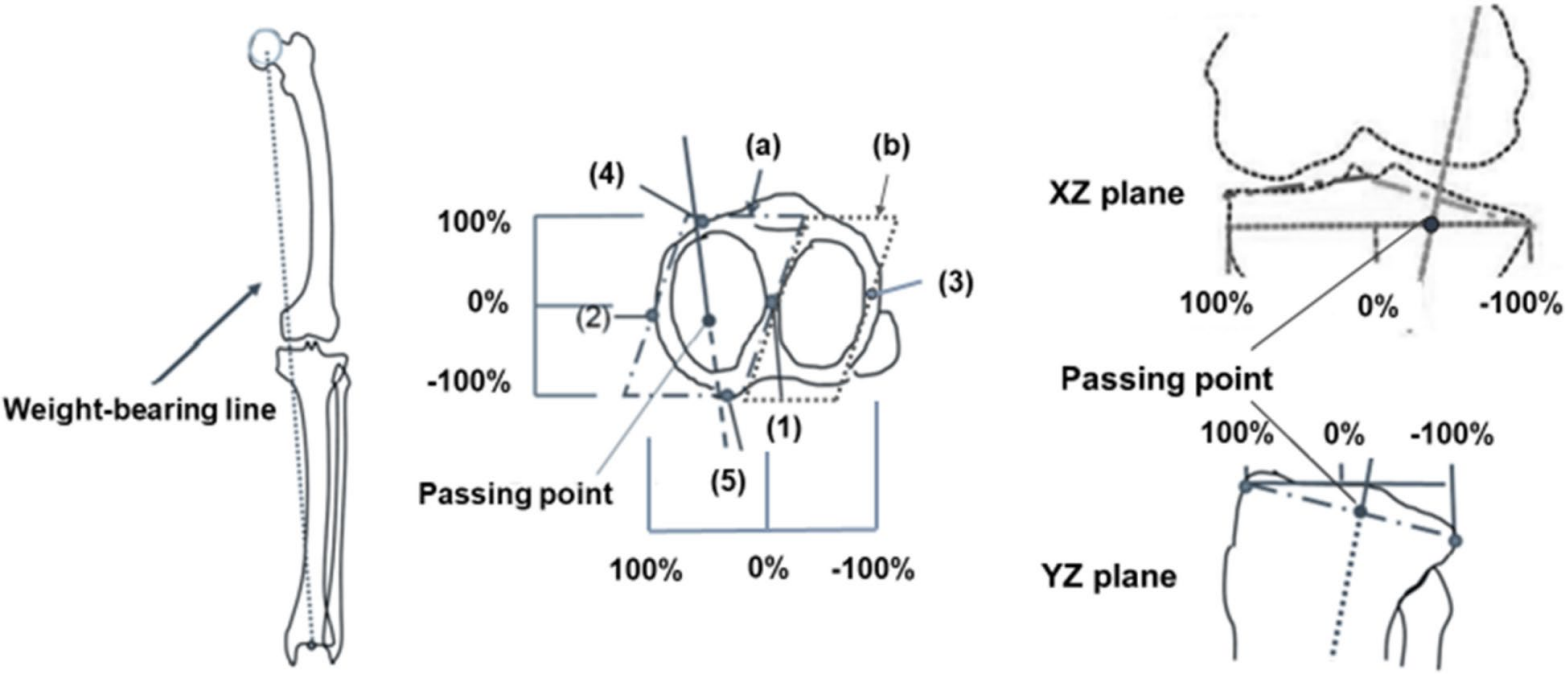
Passing point of the weight-bearing line on the tibial surface plane. (1) the origin of the tibial coordinate system, (2) the most medial point on the medial tibial surface, (3) the most lateral point on the lateral tibial surface, (4) the most anterior point on the tibial surface, (5) the most posterior point on the tibial surface of the medial and lateral tibial surface planes. The medial and lateral tibial surface planes consist of (1),(2),(4),(5) and (1),(3),(4),(5), respectively

The 3D WB line was defined as the line connecting the femoral head with the center of the ankle joint. The passing point (PP) of the WB line was defined as the location on the tibial surface plane in 3D space. The PP in the tibial coordinate system was described by percent indication: 0% indicates the center between the most anterior, posterior, medial and lateral point; + 100% indicates the most anterior or medial point and − 100% indicates the most posterior or lateral point on the tibial surface in 3D space. Negative percentages indicate a posterior or lateral location relative to the origin of the tibial coordinate system (Fig. 6).

Accuracy and reproducibility in determining the previously mentioned variables have been determined by previous studies [16, 19].

### Motion analysis of knee flexion

Knee motion was recorded via a flat panel detector (AXIOM Artis® dTA; Siemens) when subjects squatted from a standing position (knee fully extended) to maximum flexion. There was no control for patients’ feet positioning during dynamic knee testing. The sampling frequency was 15 frames per second, the image size was 360.2 mm and the resolution was 1512 × 1512 pixels. The mean duration of complete flexion cycle of the knee was 2.4 s. On average, the knee flexed at a rate of 51.7° per second. After the contours of the femur and tibia were manually detected in each image, the relative 3D positions of the femur and tibia in each fluoroscopic image (KneeMotion®; LEXI) were determined with a 3D-to-2D technique using an automated shape-matching algorithm [15]. The relative motion between the femur and tibia was obtained by performing this procedure for all images. The root mean square error was 0.3–0.8 mm for inplane translation, 2.2 mm for out-of-plane translation and 0.2°–0.6° for rotation [17]. The rotation angle of the femur relative to the tibia was defined according to the definitions reported by Grood et al. [20]. Intra- and inter-observer reliability of our 3D-to-2D image registration technique was examined via intra-class correlation coefficient (ICCs) in a previous study. The ICC (1, 2) for the rotation angle of the axis was 0.98 and the ICCs for the AP translation for the medial and lateral ends of the axis were 0.91 and 0.85, respectively. The ICC (2, 1) for the rotation angle of the axis was 0.92 and the ICCs for the AP translation of the medial and lateral ends of the axis were 0.86 and 0.99, respectively [21].

### Evaluation parameters

The following parameters were measured: (1) the inclination angle of the lateral posterior condyles in the coronal plane of the femoral coordinate system, (2) the inclination angle of the medial posterior condyles in the coronal plane of the femoral coordinate system, (3) the medial/lateral ratio of the radii of the posterior condyles approximated as spheres, (4) the spherical condylar angle, (5) the posterior condylar angle, (6) medial PTS, (7) lateral PTS, (8) the difference of medial and lateral PTS, (9) the tibiofemoral rotation angle, (10) the 3DFTA in the coronal plane, (11) the 3DHKA in the coronal plane, (12) the PP of the WB line (ML), and (13) the PP of the WB line (AP). In addition, the relationships between the rotation angle of the femur, as determined by motion analysis of the knee flexion and the previous mentioned parameters were examined.

### Statistical analyses

Correlations between the rotation angle of the femur and the measured parameters were evaluated using correlation coefficients. The distribution was examined using Shapiro–Wilk tests. The correlations were evaluated using the Pearson product-moment correlation when the data had a normal distribution and the Spearman’s rank–order correlation when the data did not have a normal distribution. The influence of the correlated parameters on the external rotation angle of the femur was calculated using multiple linear regression analysis. The statistical significance level was set at *p* < 0.05. Statistical analyses were performed using statistical software (IBM SPSS statistics version 27; IBM Corp., Armonk, NY, USA).

For the sample size calculation (α error: 0.05, 1 − β error: 0.8, correlation 0.506), 28 knees were required to evaluate the main result of the correlation between the rotation angle of the femur and parameters of bone morphology and alignment of the lower extremities. The sample size for this study was adequate (41 knees).

## Results

The mean age of the subjects was 68 ± 4 years (range, 61–76 years), the mean height was 163 ± 7 cm (149–177 cm), the mean body weight was 59 ± 10 kg (41–75 kg) and the mean body mass index was 22.1 ± 2.4 kg/m^2^ (18.3–26.0 kg/m^2^) (Table 1).

**Table 1:** The demographic data

Demographic		
Number of knees: Male/Female	28/13	
	Mean ± SD	95%CI
Age, years	68 ± 4	67–69
Body height, cm	163 ± 7	161–165
Body weight, kg	59 ± 10	57–62
Body mass index, kg/m^2^	22.1 ± 2.4	21.5–22.7

### Mean values of each parameter

**Fig. 7:**
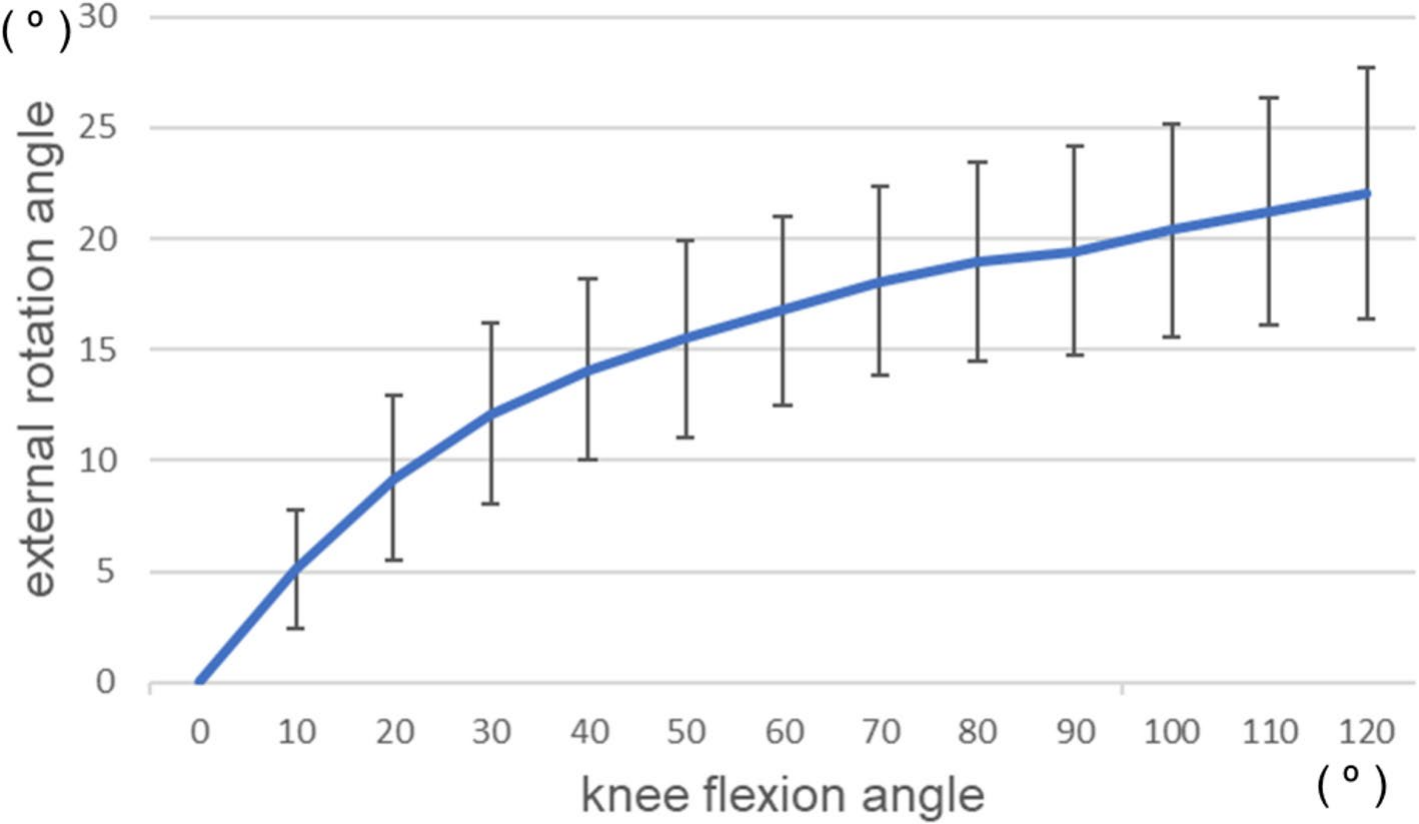
External rotation angle of the femur throughout knee flexion. The femur was substantially rotated up to 20° of knee flexion, and gently rotated after 20° of knee flexion

The external rotation angle of the femur is shown in Fig. 7. The range of knee flexion angles (0°–120°) obtained from all subjects was evaluated. The mean external rotation angle was 22.0 ± 5.7° for the 0°–120° knee flexion. The femur externally rotated substantially in early knee flexion phase and gently rotated thereafter. The inflection point of the knee rotation angle was analyzed using linear approximation with 0–10 degrees and 30–120 degrees, and was determined to be 24.7 degrees of knee flexion. After 24.7 degrees, the change in the rotation angle of the knee became gradual. Thus, correlations for each parameter were evaluated separately for the 0°–20° and 20°–120° knee flexion angles. The mean values of the parameters are shown in Table 2.

**Table 2:** Evaluation parameters

Parameters		
	Mean ± SD	95%CI
External rotation angle		
0–20° knee flexion	9.2 ± 3.7	8.0–10.4
20–120° knee flexion	12.8 ± 6.2	10.9–14.8
0–120° knee flexion	22.0 ± 5.7	20.2–23.8
Femoral morphology		
LFC inclination angle	7.8 ± 7.2	5.6–10.1
MFC inclination angle	0.8 ± 5.9	-1.1–2.7
Ratio of radii of posterior condyles	1.0 ± 0.1	1.0–1.0
Spherical Condylar Angle	1.8 ± 1.0	1.5–2.1
Posterior Condylar Angle	3.1 ± 1.3	2.7–3.5
Tibial morphology		
Medial PTS	9.9 ± 2.5	9.1–10.7
Lateral PTS	6.8 ± 3.2	5.8–7.8
Difference of medial and lateral PTS	3.1 ± 3.1	2.1–4.0
Alignment		
Tibiofemoral rotation angle	8.6 ± 6.6	6.8–10.4
3DFTA in coronal plane	178.1 ± 2.5	177.4–179.0
3DHKA in coronal plane	182.1 ± 2.5	181.4–182.8
PP of the WB line < ML >	27.6 ± 19.9	21.3–33.8
PP of the WB line < AP >	-7.4 ± 82.9	-33.6–18.8

*LFC* Lateral femoral condyle, *MFC* Medial femoral condyle, *PTS* Posterior tibial slope

*FTA* Femorotibial angle, *HKA* Hip knee ankle angle, *PP* Passing point

*WB* Weight bearing, *ML* Medial–lateral; AP = Anterior–posterior

### Correlation with external rotation of the femur and femoral and tibial morphology and alignment

No significant correlations were detected between any parameters during 0°–20° of knee flexion (Table 3). The external rotation angle correlated with the spherical condylar angle (correlation coefficient, − 0.506; *p* = 0.001), the tibiofemoral rotation angle (correlation coefficient, 0.364; *p* = 0.019), and the inclination angle of the medial posterior condyles (correlation coefficient, 0.337; *p* = 0.031) during 20°–120° of knee flexion (Table 3). A multiple linear regression was conducted to predict the external rotation angle of the femur based on spherical condylar angle, tibiofemoral rotation, and MFC inclination angle. The regression analysis revealed a significant equation (F (3, 37) = 10.006, *p* < 0.000), with an adjusted R squared of 0.403. The regression model predicted that the external rotation angle of the femur is determined by the following linear regression equation: 16.406 – 3.117 (spherical condylar angle) + 0.256 (tibiofemoral rotation) – 0.324 (MFC inclination angle). In this equation, spherical condylar angle is measured in degrees, tibiofemoral rotation is measured in degrees, MFC inclination angle is measured in degrees. A one-unit increase in spherical condylar angle corresponded to a change of 3.117 the external rotation angle of the femur, a one-unit change in tibiofemoral rotation corresponded to a change of 0 0.256 the external rotation angle of the femur, and a one-unit change in MFC inclination angle corresponded to a change of 0 0.324 the external rotation angle of the femur. Spherical condylar angle, tibiofemoral rotation and MFC inclination angle demonstrated significant predictive power for the external rotation angle of the femur. Multiple regression analysis results show that the spherical condylar angle was the most influential in correlated parameters with femoral external rotation (Table 4). External rotation of the femur correlated with the spherical condylar angle during 20°–120° of knee flexion but did not correlate with the inclination angle of the posterior lateral condyles.

**Table 3:** Correlation coefficient for axial rotation

	External rotation			
	0–20°		20–120°	
Parameters	*r*	*p* value	*r*	*p* value
LFC inclination angle	-0.178	n.s	0.120	n.s
MFC inclination angle	0.026	n.s	-0.337	.031*
ratio of radii of posterior condyles	-0.067	n.s	-0.011	n.s
Spherical Condylar Angle	0.101	n.s	-0.506	.001*
Posterior Condylar Angle	-0.154	n.s	-0.106	n.s
Medial PTS	-0.239	n.s	0.241	n.s
Lateral PTS	0.003	n.s	0.243	n.s
Difference of medial and lateral PTS	0.201	n.s	0.057	n.s
Tibiofemoral rotation	0.018	n.s	0.364	.019*
3DFTA in coronal plane	0.226	n.s	0.153	n.s
3DHKA in coronal plane	0.292	n.s	-0.011	n.s
PP of the WB line < ML >	0.167	n.s	0.079	n.s
PP of the WB line < AP >	-0.070	n.s	-0.180	n.s

*LFC* Lateral femoral condyle, *MF*C Medial femoral condyle, *PTS *Posterior tibial slope

*FTA* Femorotibial angle, *HKA* Hip knee ankle angle, *PP* Passing point

*WB* Weight bearing, *ML* Medial–lateral, *AP* Anterior–posterior

^*^Significant difference = *p* < 0.05

**Table 4:** Results of multiple regression analysis

Independent variable	B	SE	β	t	*p* value	95%CI
Spherical condylar angle	3.12	0.780	0.49	3.99	< .001	1.54 – 4.70
Tibiofemoral rotation	0.26	0.120	0.27	2.14	.039	0.01 –0.50
MFC inclination angle	-0.32	0.130	-0.31	-2.49	.018	-0.59 – -0.06

*SE* Standard error, *CI* Confidence Interval, adjusted *r*-square: 0.403

Dependent variable: external rotation angle of the femur

## Discussion

The most important finding of the present study was that the spherical condylar angle correlated with the rotation angle of the femur relative to the tibia at 20°–120° of knee flexion. In this study, the femur was substantially rotated externally from extension to 20° of knee flexion, followed by gradual external rotation up to about 120° of flexion. This trend was similar to previous studies [1, 5, 21]. The purpose of this study was to identify factors related to the external rotation angle.

**Fig. 8:**
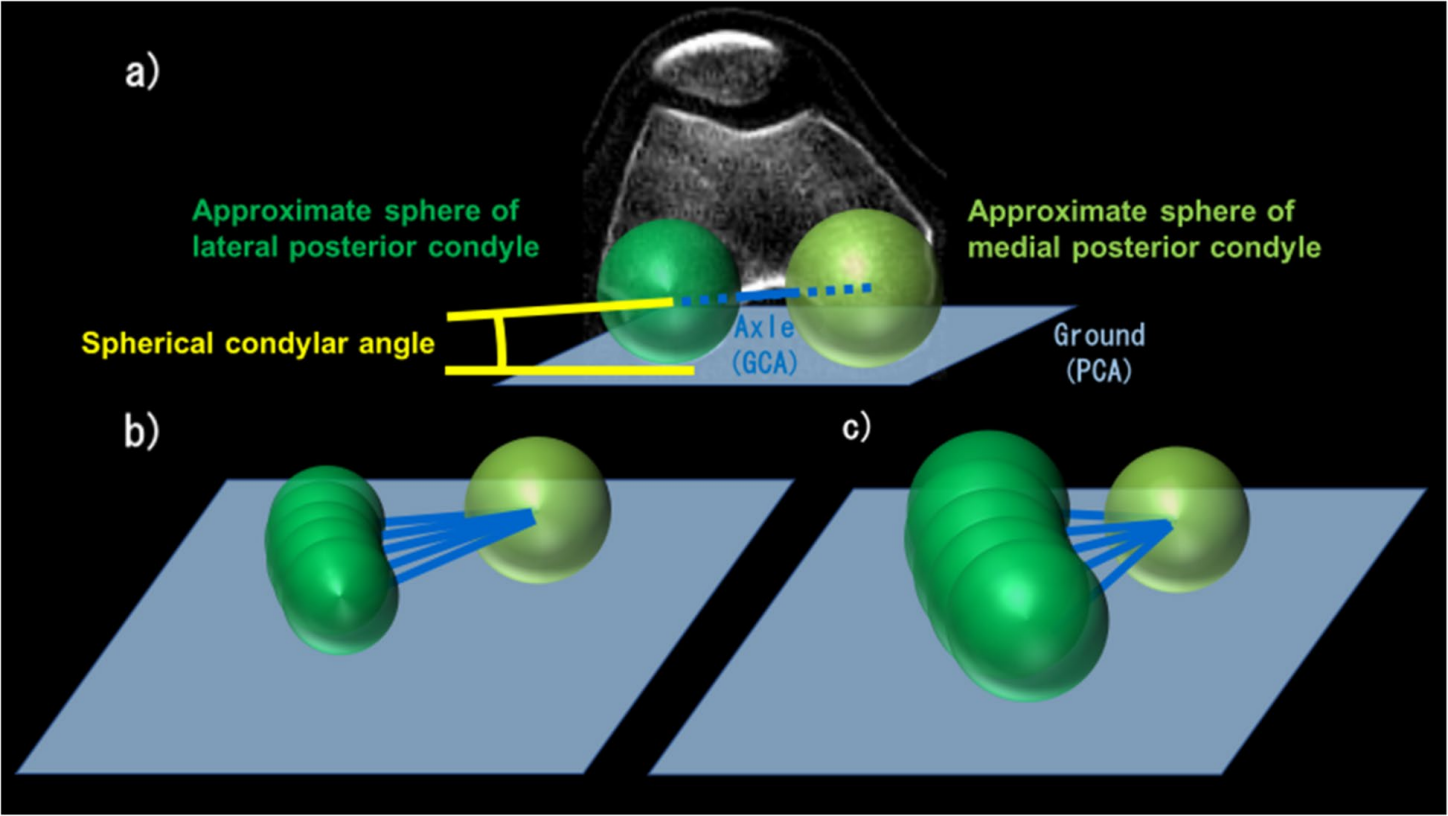
Relationship between spherical condylar angle and rotation angle. **a** The Spherical condylar angle can be likened to the angle between the axle and the ground. If the changed in knee flexion angle is the same, **b**) rotation angle is small when the approximate sphere of lateral posterior condyle is small. **C** rotation angle is large when the approximate sphere of medial posterior condyle is big

The rotation angle of the femur significantly correlated with the spherical condylar angle during 20°–120° of knee flexion. The spherical condylar angle is the angle between the GCA and PCA that is tangent to the tibial articular surface. If the GCA is the approximate flexion–extension axis of the knee joint during mid-flexion to deep flexion, GCA is the axle connecting two differently sized spheres of the medial and lateral posterior condyles of the femur, and PCA is the ground. Regarding the rolling motion of the femur relative to the tibia excluding sliding and slipping motion of the femur, the rotation angle of the femur per the same flexion angle depends on the size of medial and lateral approximate spheres during medial pivot motion (Fig. 8). The spherical condylar angle is influenced by these sizes, and thus exhibited a correlation with the femoral rotation angle. However, the rotation angle of the femur did not correlate with the ratio of the posterior condyle radii. In addition to the size of the posterior condyles, the spherical condylar angle is influenced by the distance between the center of approximate sphere of medial and lateral posterior condyles. On the other hand, the ratio of the posterior condyle radii is influenced only by the posterior condyle sizes. In other words, the radii ratio does not directly affect the GCA, which is the approximate flexion–extension axis. Therefore, the ratio of the posterior condyle radii did not correlate with the rotation angle of the femur. Hoshino et al. performed in vivo motion analysis to evaluate knee kinematics during downhill running on a treadmill using a 3D to 2D image registration technique. They showed that the internal tibial rotation positively correlated with the condylar twist angle, although they defined the condylar twist angle as the angle between the trans-epicondylar angle and transcondylar angle, which is the same as the GCA [22]. Thus, they found that the morphological characteristics of the femoral condyle, which were associated with the approximated flexion–extension axis, affected the tibiofemoral rotation. The correlation between the rotation angle of the femur and the flexion–extension axis of the knee was also demonstrated in our study, although the analyzed motions were different in the two studies.

**Fig. 9:**
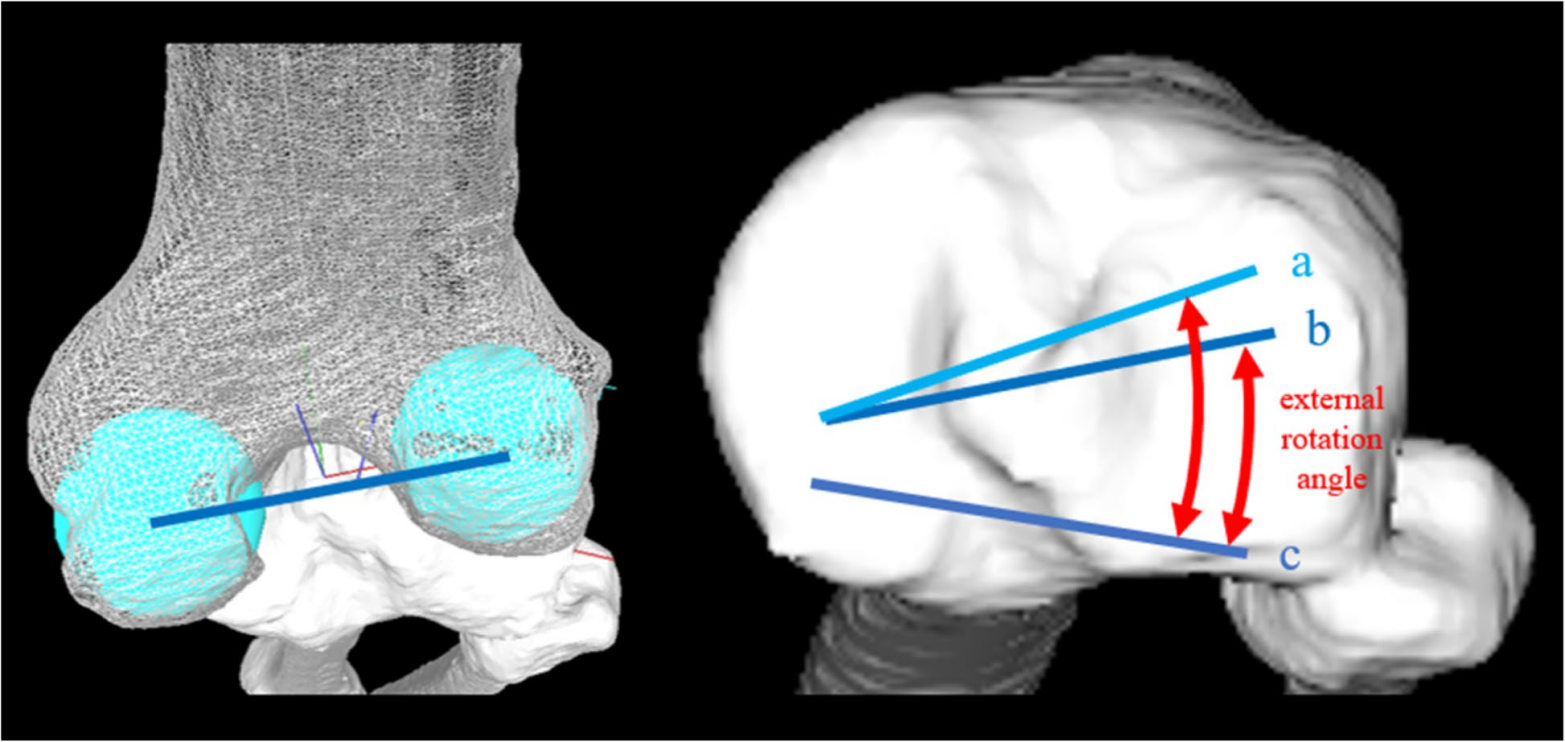
The relationship between the tibiofemoral rotation angle and the femoral rotation angle. Lines a and b depict the geometric center axis (GCA) in knee extension. Line c depicts the GCA in full knee flexion. As the femur becomes more internally rotated relative to the tibia in the knee extension (line a > line b), the external rotation angle of the femur increases

In this study, the tibiofemoral rotation angle was also correlated with the femoral rotation angle relative to the tibia throughout 20°–120° of knee flexion. If tibiofemoral rotation angle under the WB condition was considered as equivalent to the initial tibiofemoral rotation angle during the motion analysis, the femur with more internal rotation relative to the tibia in the WB position increased the femoral external rotation angle during knee flexion, implying that the initial internal rotation of the femur may provide the femur more space to externally rotate (Fig. 9).

**Fig. 10:**
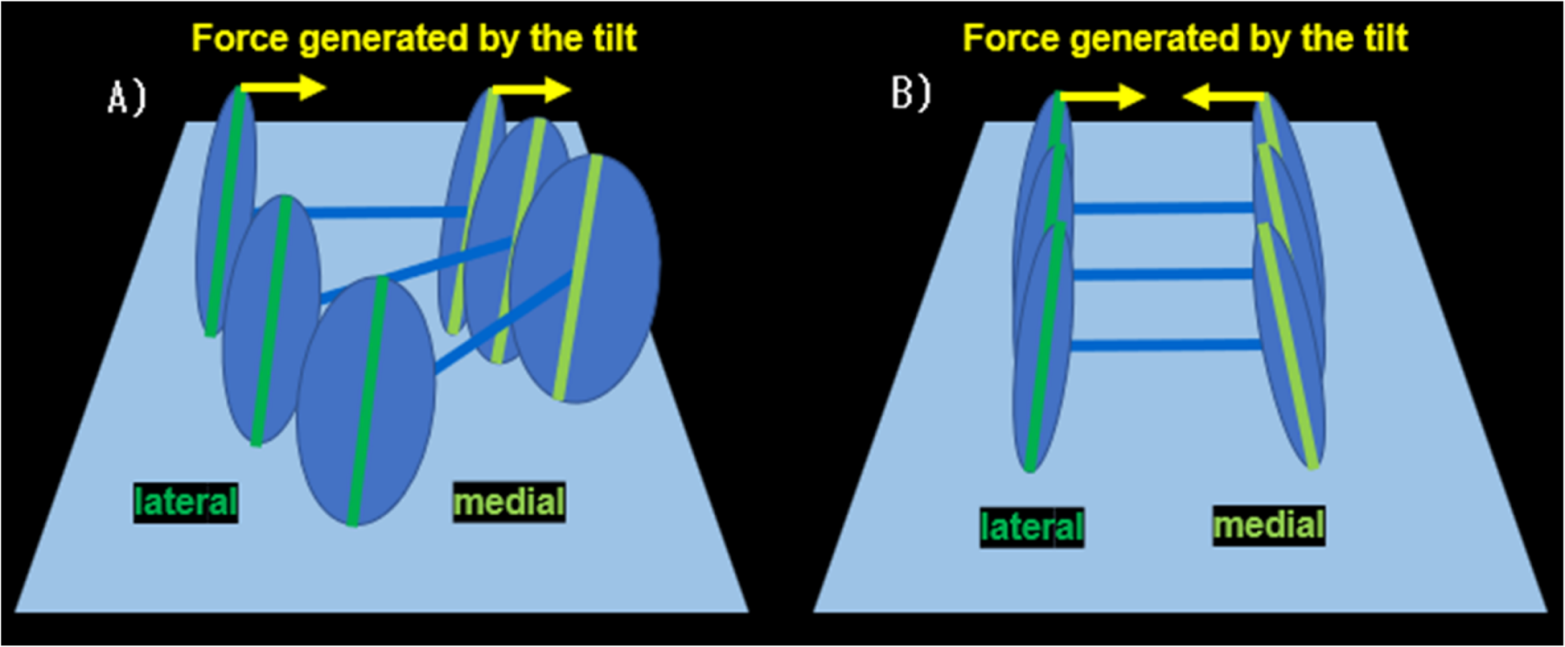
The relationship between the tilt of posterior condyle and the rotation of the femur. **A** When both the medial and lateral posterior condyles are tilted medially, a medially directed force is produced during knee flexion, resulting in external rotation of the femur. **B** When the medial posterior condyle is tilted laterally, the forces generated by the tilt of medial and lateral posterior condyles are cancelled out

Furthermore, the inclination angle of the medial posterior condyles was correlated with the rotation angle of the femur relative to the tibia throughout 20°–120° of knee flexion in this study. When the medial posterior condyle tilted medially (in the same direction as the lateral posterior condyle), the external rotation angle of the femur was increased. If the fulcrum of the pivot motion, the medial posterior condyle, has an opposite tilt relative to the lateral posterior condyle, it may create an antagonistic force to the lateral posterior condyle moving medially (i.e., externally) during knee flexion. Conversely, it was thought that if the medial posterior condyle had the same directional inclination as the lateral posterior condyle, the medial translation (i.e., externally rotation) of the lateral posterior condyle could be induced smoothly (Fig. 10).

No correlations between any parameters were detected during 0°–20° of knee flexion. This may be partially due to the effect of the anterior cruciate ligament (ACL). The femoral external rotation up to 20° of knee flexion is the same as the screw-home movement [23, 24]. Several reports demonstrate that the screw-home movement is induced by the ACL. Using MRI analysis, Ng et al. demonstrated the absence of normal screw-home movement with relative anterior movement of the lateral tibial condyle between 30° of flexion and full extension in 51% of patients with ACL tears [25]. Morishige reported that the knee external rotation (the external rotation of the tibia relative to the femur during knee extension from a flexed position) angle was significantly smaller on the ACL tear side compared with the angle on the contralateral unaffected side during 0°–15° of knee flexion [26]. Factors, such as the ACL, may play a crucial role in tibiofemoral movement up to 20°, but these factors may have less influence during 20°–120° of knee flexion. The lack of correlation between any parameters during 0°–20° of knee flexion may also be due to the flexion–extension axis of the knee, which is approximated by the GCA at around 90° of knee flexion. However, from extension to the early flexion phase, another approximate flexion–extension axis may exist. Using bone morphometric analysis of the femoral condyle, Sato et al. reported that the SEA was an approximate flexion–extension axis during 0°–80° of knee flexion [18]. Feng et al. reported the constant vertical positions of SEA and GCA during 0°–60° and 30°–90° of knee flexion, respectively [1]. Several reports suggest a transition of the flexion–extension axis of the knee [2, 4]. The lack of correlation between the rotation angle of the femur and the spherical condylar angle can be explained by a transition of the flexion–extension axis from GCA to SEA during early flexion to extension.

The previous study reported that the magnitudes of axial rotation decreased after TKA when compared to normal knee patients during a deep knee bend maneuver, and axial rotation provides a mechanical advantage in deep knee flexion and is thus desirable in TKA [9, 27]. In current study, the spherical condylar angle correlated with the rotation angle of the femur relative to the tibia during 20°–120° of knee flexion. As a result, the concept of spherical condylar angle could be incorporated into TKA design.

Several limitations should be noted in the current study. First, our study evaluated bone morphology and did not include cartilage. However, the medial and lateral femoral condyle cartilage thicknesses are not different [28]. At the contact points during 30°–90° of knee flexion, the maximum differences in medial and lateral cartilage thicknesses were 0.3 mm and 0.6 mm, respectively [29]. Based on these studies, the effects of cartilage on the spherical condylar angle are not likely to be large. Second, our study did not include flexion angles over 120° of deep flexion. However, the femur hardly rotates externally during knee flexion of over 120° and moves posteriorly (bicondylar rollback) [5, 30]. Therefore, regarding the rotation of the knee, evaluation of tibiofemoral rotation might be enough without analyzing over 120° of deep flexion. Third, the results of our study were obtained from Japanese subjects may be different from the results obtained from other races. Further studies about the racial difference of bone morphology and motion analysis will be needed. Force, the motion of femur relative to the tibia was considered only the rolling motion of the femur in this study. In the fact, the femoral motion relative to the tibia was not only a rolling motion, but also combination of sliding and slipping motions. Thus, further detailed analysis of femoral motion relative to the tibia is desirable.

## Conclusion

In this study several morphological features were found to be correlated with the rotation angle of the femur relative to the tibia, with a particularly significant correlation observed with the spherical condylar angle. In TKA, morphology plays a significant role in defining motion, and the findings of this study provide useful information, which may be applied in the design of TKA.

## Data Availability

Data will be made available on reasonable request.

## Abbreviations

ACL: Anterior cruciate ligament
CT: Computed tomography
GCA: Geometric center axis
ICC: Intra-class correlation coefficient
PCA: Posterior condylar axis
PP: Passing point
PTS: Posterior tibia slope
SEA: Surgical epicondylar axis
WB: Weight-bearing
